# Assessing modifiable risk factors for atrial fibrillation/flutter in the young: a hybrid local-global study

**DOI:** 10.3389/fendo.2026.1806013

**Published:** 2026-05-29

**Authors:** Ye Liu, Lifeng Liu, Qing Zhou, Yupeng Liu, Jingjing Song

**Affiliations:** 1 Heart Center and Beijing Key Laboratory of Hypertension, Beijing Chaoyang Hospital, Capital Medical University, Beijing, China; 2 Department of Cardiology, Beijing Hospital, National Center of Gerontology, Institute of Geriatric Medicine, Chinese Academy of Medical Sciences, Beijing, China; 3 Department of Cardiology, Guangdong Provincial People’s Hospital (Guangdong Academy of Medical Sciences), Southern Medical University, Guangzhou, China; 4 Guangdong Cardiovascular Institute, Guangdong Provincial People’s Hospital (Guangdong Academy of Medical Sciences), Guangzhou, China

**Keywords:** atrial fibrillation, atrial flutter, high blood pressure (hypertension), risk factor, young population

## Abstract

**Background:**

Atrial fibrillation (AF)/atrial flutter (AFL) are among the most prevalent cardiovascular diseases in the young. This study aimed to assess modifiable risk factors by integrating a global data analysis with a local cohort study.

**Methods:**

Age-standardized rates of AF/AFL prevalence (ASPR), incidence (ASIR), mortality (ASDR), and DALYs, EAPCs were analyzed by regions from 1990 to 2021. In the cohort study, multivariable logistic regression analysis was performed to evaluate the association between risk factors and the odds of atrial arrythmia. Restricted cubic splines (RCS) were used to explore potential non-linear relationships between risk factors and the odds of atrial arrhythmia.

**Results:**

A rising global burden of AF/AFL was observed among individuals aged 15 to 39 years. High-SDI areas had the highest AF/AFL-related ASPR, ASIR, and DALYs. Global data identified hypertension, high BMI, smoking, and alcohol consumption as the main modifiable risk factors for AF/AFL, among which hypertension contributes the most. In the cohort study, segmented regression identified significant breakpoints for DBP at 88.3 mmHg and BMI at 28.8 kg/m^2^, while SBP was positively associated with AF above an estimated value of 117.0 mmHg without a statistically significant slope-change test. Bayesian age-period-cohort (BAPC) analysis projected a modest decline in AF/AFL burden among the young over the next 3 decades.

**Conclusion:**

The global burden of AF/AFL in the young adult population has increased over the past 30 years. High blood pressure, high BMI, smoking, and alcohol consumption are key modifiable contributors to the increased AF/AFL burden in young adults. Enhanced awareness, targeted screening, and proactive clinical management of these risk factors are critical for mitigating the AF/AFL burden in this demographic.

## Introduction

Atrial fibrillation and atrial flutter (AF/AFL) are the most prevalent cardiac arrhythmias globally, affecting over 60 million people worldwide in 2019 and projected to double in burden by 2050 (1). AF/AFL impairs life quality of the patients effectively and leads to an increased susceptibility to heart failure, stroke, cardiac arrest and other complications (2–6). In 2019, an estimated 315337 deaths were attributed to AF/AFL worldwide.¹ Between 1990 and 2019, the age-standardized death rate for AF/AFL increased by 2%, emerging substantial health and financial burden to the world (1).

Since the prevalence of AF/AFL has traditionally been associated with aging populations, prior research has predominantly focused on elderly population (7). Recent studies, however, demonstrate a concerning increase in AF/AFL among individuals aged 15 to 39 years, potentially attributable to rising rates of hypertension, metabolic disorders, occupational stress, tobacco use, and alcohol consumption (1, 8, 9). Although adults older than 65 years continue to exhibit the highest overall AF/AFL prevalence, both the prevalence and incidence rates have risen steadily in younger cohorts (8, 10). In the population aged 15–39 years, atrial fibrillation and flutter was the most prevalent cardiovascular disease with the age-standardized prevalence rate (ASPR) of 7.85 and age-standardized incidence rate (ASIR) of 2.36 globally in 2019 (8).

Young people constitute the backbone of the global labor force, with their health status directly impacting long-term economic stability. Recent evidence demonstrates a concerning rise in AF/AFL among younger populations, associated with modifiable risk factors including sedentary behaviors, suboptimal nutrition, and substance use (11, 12). Despite these observations, comprehensive epidemiological data remain limited. The awareness of AF/AFL in young population is limited and frequently delayed, as symptoms may be misinterpreted as manifestations of anxiety or other benign conditions. Unaware and untreated AF/AFL in this demographic contributes to diminished workforce participation and productivity, underscoring the urgent need for targeted public health initiatives focused on prevention and early management of cardiovascular conditions in the young (12, 13).

This study aimed to: (1) uncover the epidemiological trends of AF/AFL and attributable risk factors in the young population aged 15–39 years from 1990 to 2021, stratified by sex, region, country, and sociodemographic index (SDI); (2) explore nonlinear risk factor-disease relationships for AF/AFL and identify specific clinical intervention threshold based on global and local cohort data; (3) project global ASPR and DALYs burdens through 2050 with Bayesian age-period-cohort (BAPC) modeling. This study will provide critical evidence to inform future evidence-based guidelines, prevention strategies, and disease control measures for AF/AFL in young populations.

## Methods

### Data acquisition

The GBD 2021 study employed the latest epidemiological data to evaluate the health loss of 369 diseases, injuries, and impairments with 88 risk factors around worldwide 204 nations and territories. Detailed study design and methods have been described in previous literatures (14, 15). This study adhered to the Guidelines for Accurate and Transparent Health Estimates Reporting (GATHER) (16). And the University of Washington Institutional Review Board waived the requirement for informed consent to access the GBD data (17).

### Estimation framework

The GBD study estimated the worldwide burden of AF/AFL with sophisticated modeling techniques. The incidence and prevalence of AF/AFL was calculated with DisMod-MR 2.1 (disease-model-Bayesian meta- regression) software, which incorporated diverse disease parameters, epidemiological relationships and geospatial data to conduct robust estimates. The Cause of Death Ensemble modeling (CODEm) framework was applied to estimate mortality. Diverse models were applied to 2021 data to estimate AF/AFL prevalence, incidence and mortality with improved precision. We further calculated DALYs due to AF/AFL by summing the Years Lived with Disability (YLD) and the Years of Life Lost (YLL).

### Sociodemographic index

The SDI comprehensively evaluates the development level of a country/region with fertility rate, education level, and per capita income data (18). The SDI ranges from 0 to 1, the higher the SDI is, the greater the socioeconomic development is. In this study, countries and regions were classified into five SDI categories (low, low-medium, medium, medium–high, and high) to evaluate the association between AF/AFL burden and socioeconomic development.

### Risk factors

This study examined several specific risk factors contributing to AF/AFL burden, including high systolic pressure, smoking, high alcohol use and high body-mass index (BMI). We analyzed the AF/AFL-related deaths and DALYs attributable to these risk factors and further stratify the results by regions to elucidate the influence of geographical variations. DisMod-MR 2.1 and spatiotemporal Gaussian process regression were employed to model exposure distributions for each risk factor across various demographics and locations. Then the theoretical minimum risk exposure level (TMREL) of each risk factor according to epidemiological evidence was examined, which represents the optimal exposure level for minimizing ischemic stroke risk (19). Then the population attributable fractions (PAFs) for each risk factor were calculated by integrating TMREL, relative risk estimates and exposure data and further stratified by age, sex and location. The value calculated by PAFs multiply DALYs helps to estimate the risk-attributable burden and offer valuable insight into controlling AF/AFL by modifying risk factors among different populations.

### Statistical analysis of GBD database

This study utilized the Estimated Annual Percentage Change (EAPC) to examine the trends in age-standardized rates (ASR) of AF/AFL incidence, mortality, DALYs, and prevalence.


$$\text{ASR}=\frac{\sum_{i=1}^{A}a_{i}w_{i}}{\sum_{i=1}^{A}w_{i}}\times 100,000$$

Both the EAPC and the lower bound of its 95% CI are positive indicates an increase trend of ASR, whereas when the EAPC and the upper bound of its 95% CI are negative, the ASR is deemed to have a decreasing trend. And the ASR is considered to stable when neither condition is satisfied. The relationship between SDI and age-standardized rates of AF/AFL was assessed by spearman correlation. And Bayesian age-period-cohort (BAPC) model, which exhibits better coverage and precision than other predictive models (20), was utilized to project future trends of AF/AFL burden, the R-package BAPC implements all the processes. All the analyses in this study were conducted with the R statistical computing software (Version 4.2.2).

### Study design of the cohort study

This study was a retrospective analysis of patients who underwent electrophysiological procedures at Guangdong Provincial People’s Hospital. This study was approved by the Ethics Review Committee of Guangdong Provincial People’s Hospital. Inclusion criteria for this analysis were: (1) age 39 years or younger at the time of the procedure, and (2) availability of complete baseline blood pressure (systolic and diastolic) and body mass index (BMI) data, which was systematically recorded for patients from 2020 to 2023. Finally, 1015 individuals were enrolled in this study, and then divided into a case group (either paroxysmal AF or persistent AF) and a control group (premature ventricular contractions, PVC) based on their primary diagnosis. This group was chosen as it represents a population referred for electrophysiological evaluation but without a diagnosis of AF/AFL, SVT, AVNRT, or AVRT, thus providing a suitable baseline for comparison. Patients who had a concurrent diagnosis in both the case and control definitions were excluded from the analysis to ensure the mutual exclusivity of the groups.

### Statistical analysis of the cohort study

Baseline characteristics of the case and control groups were compared. To evaluate the association between risk factors and the odds of having AF, multivariable logistic regression analysis was performed. Two models were constructed for each risk factor: Model 1 (Unadjusted): Assessed the direct association of each risk factor with AF. Model 2 (Adjusted): Adjusted for potential confounders, specifically age and history of heart failure. Odds ratios (ORs) and their corresponding 95% confidence intervals (CIs) were calculated. To explore potential non-linear relationships between continuous variables (SBP, DBP, BMI) and the odds of AF, we utilized restricted cubic splines (RCS) with three knots. The knots were placed at the 5th, 50th, and 95th percentiles of the variable’s distribution. Furthermore, to identify specific risk thresholds, segmented regression analysis was conducted for variables that exhibited a potential threshold effect in the RCS curves. The Davies test was used to assess the statistical significance of the change-point (breakpoint). The ORs for the association before and after the identified breakpoint were calculated to quantify the change in risk. All statistical analyses were performed using R software, version 4.4.0. A two-sided *p* < 0.05 was considered statistically significant for all tests.

### Informed consent and ethics

This study comprised two components with distinct ethical considerations. For the Global Burden of Disease (GBD) analysis: The GBD study utilized publicly available, de-identified data that do not contain confidential health records or personally identifiable information. The University of Washington Institutional Review Board granted exemption status for this component, and individual informed consent was waived. For the retrospective cohort study: This study was approved by the Ethics Review Committee of Guangdong Provincial People’s Hospital. Due to the retrospective nature of the study and the use of anonymized clinical data, the requirement for individual informed consent was waived by the ethics committee.

## Results

### The AF/AFL prevalence increased in the young population aged 15–39 years

**Figure 1:**
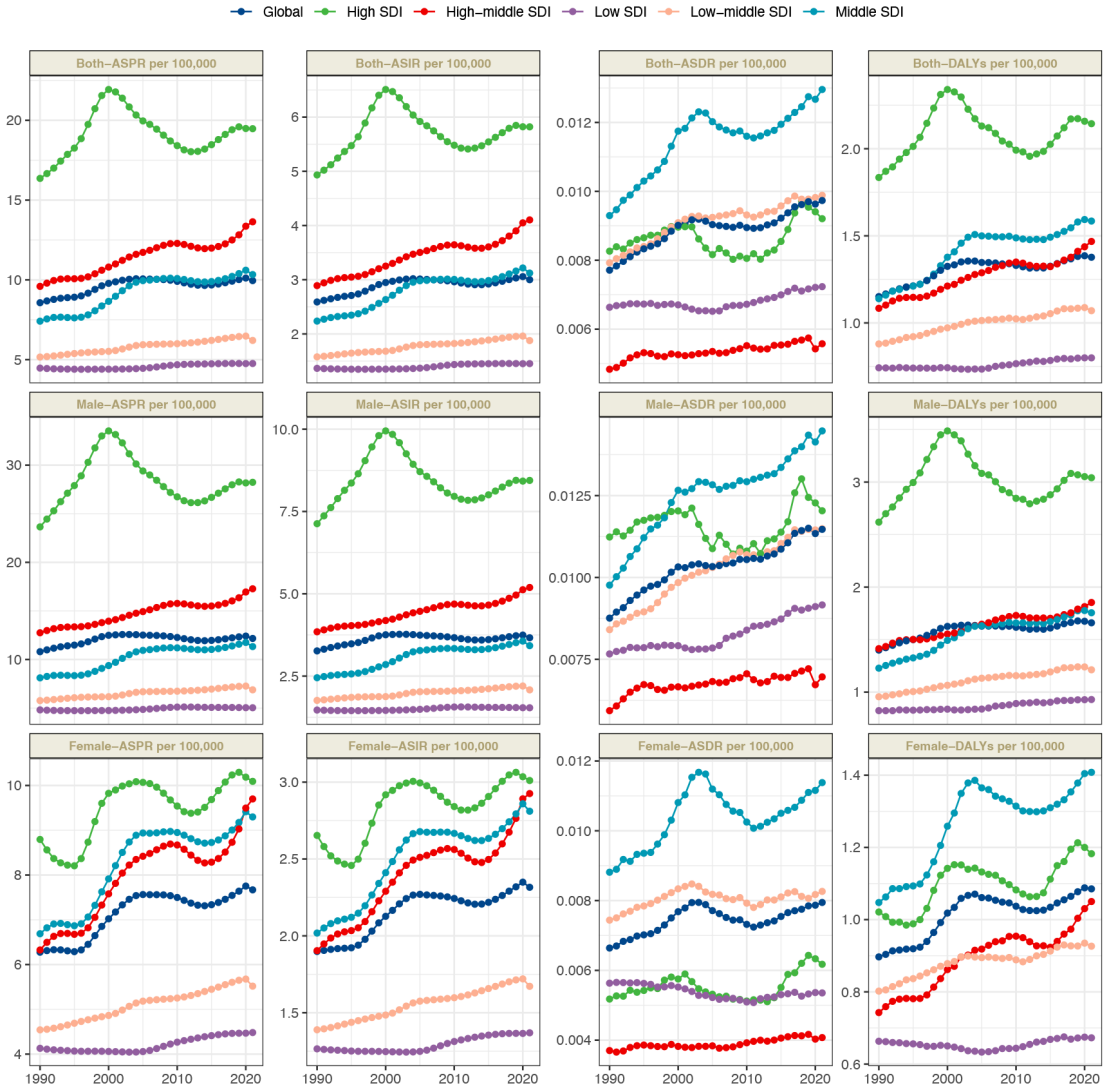
Trends in atrial fibrillation/atrial flutter prevalence, incidence, deaths and disability-adjusted life-years from 1990 to 2021.

**Figure 2:**
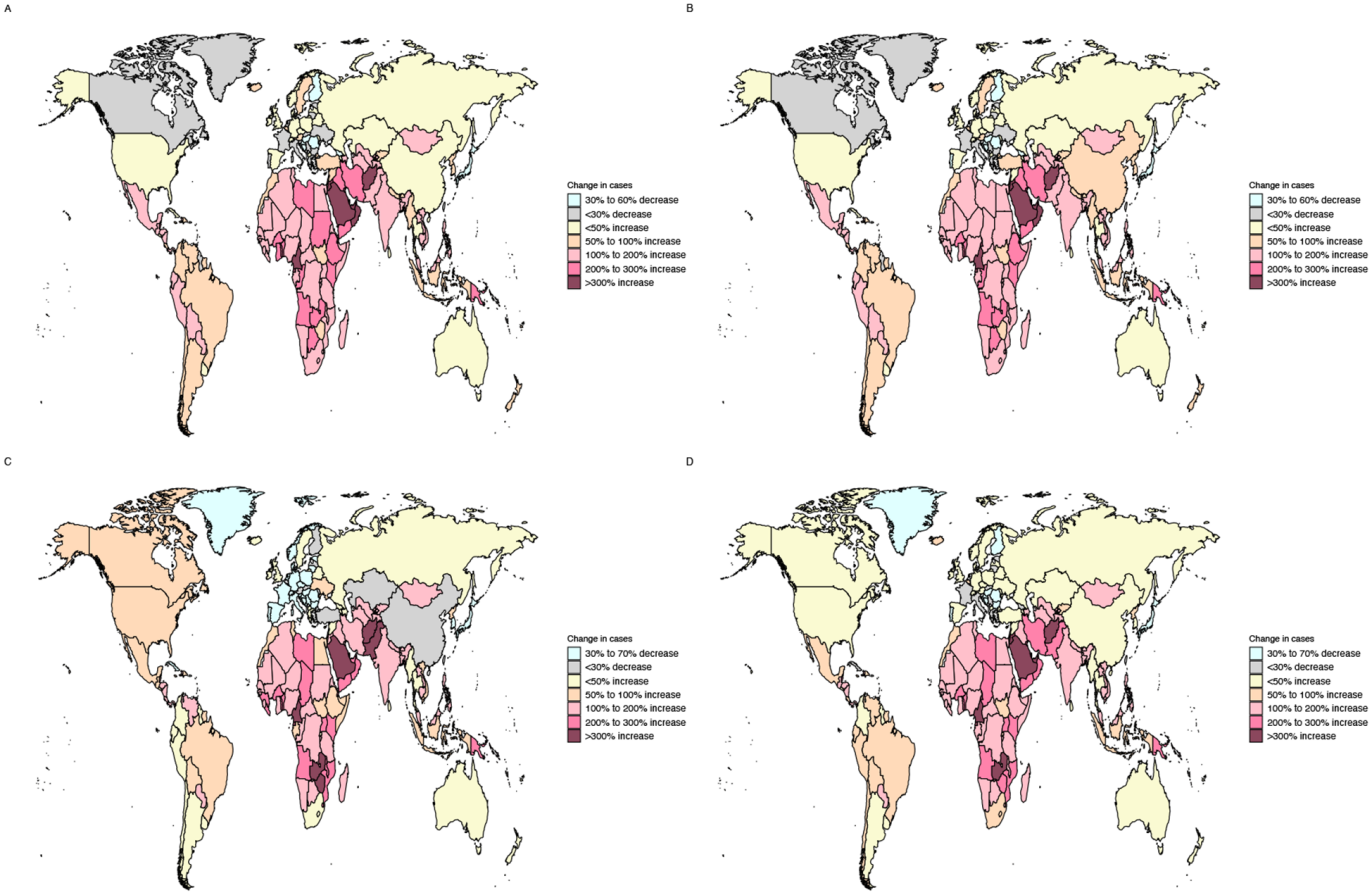
Change cases of atrial fibrillation/atrial flutter in 204 countries and territories. **(A)** Change prevalence cases. **(B)** Change incidence cases. **(C)** Change deaths cases. **(D)** Change DALYs.

In 2021, the estimated global burden of AF/AFL among population aged 15–39 years was 296062.9 cases (95% UI: 152846.3-499514.2), marking a 57.67% increase from 1990. The ASPR indicated an increase from 8.6 per 100,000 persons (95% UI: 4.1-14.9) in 1990 to 10 per 100,000 persons (95% UI: 5.1-16.8) in 2021 (**Figure 1**, **Supplementary Table 1**). The global incidence, mortality and DALYs of AF/AFL in 2021 also represented staggering increases from 1990 (**Figure 1**, **Supplementary Table 1**). The global burden of AF/AFL exhibits significant regional variations, closely tied to SDI levels. The high-SDI regions reached ASPR at 19.5 per 100,000 people (95% UI: 12.6-28) in 2021, and the highest ASIR and DALYs at 4.9 per 100,000 people (95% UI: 2.7-8.2) and 1.8 per 100,000 people (95% UI: 1.1-3) respectively (**Figure 1**, **Supplementary Table 1**). The ASPR, age-standardized DALY rates increased exponentially with SDI, which may result from the improving AF/AFL awareness, increasing hypertension prevalence and stress levels with SDI increasing (**Supplementary Figures S2**). The changes of ASPR, ASIR, ASDR and DALYs across different nations between 1990 and 2021 could be observed in **Figure 2**. The United Arab Emirates experienced the most dramatic increase in prevalence cases. Additionally, the ASIR of AF/AFL among 15-39-year population exhibited an exponential growth between age 30–39 in both males and females, and continuously higher in male compared to female within the same age group (**Supplementary Figures S3**).

### Risk factors contributing to AF/AFL in the population aged 15–39 years

**Figure 3:**
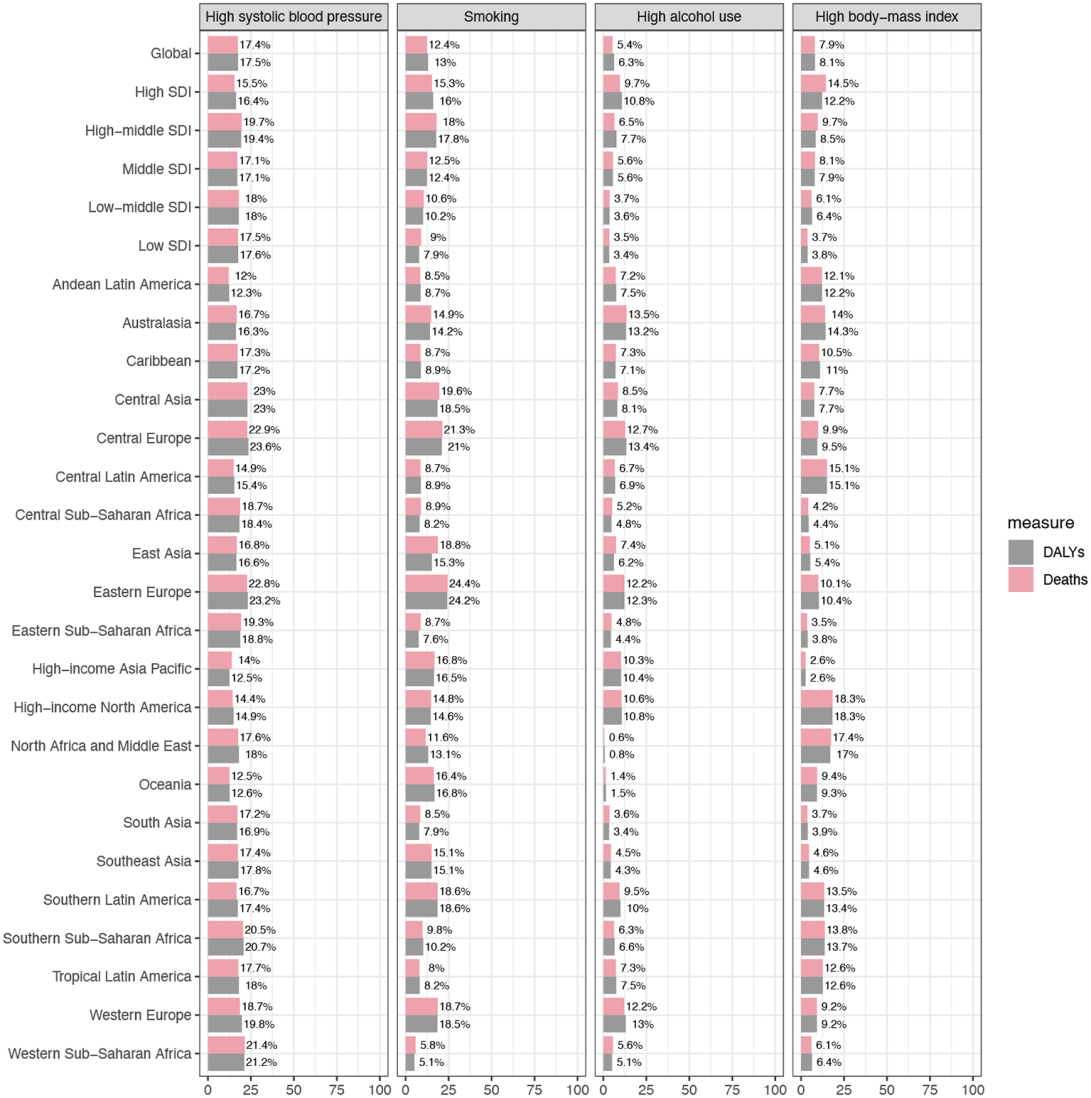
Percentage contributions of major risk factors to age-standardized death and DALYs of atrial fibrillation/atrial flutter stratified by regions and socio-demographic index, 1990-2021.

**Figure 4:**
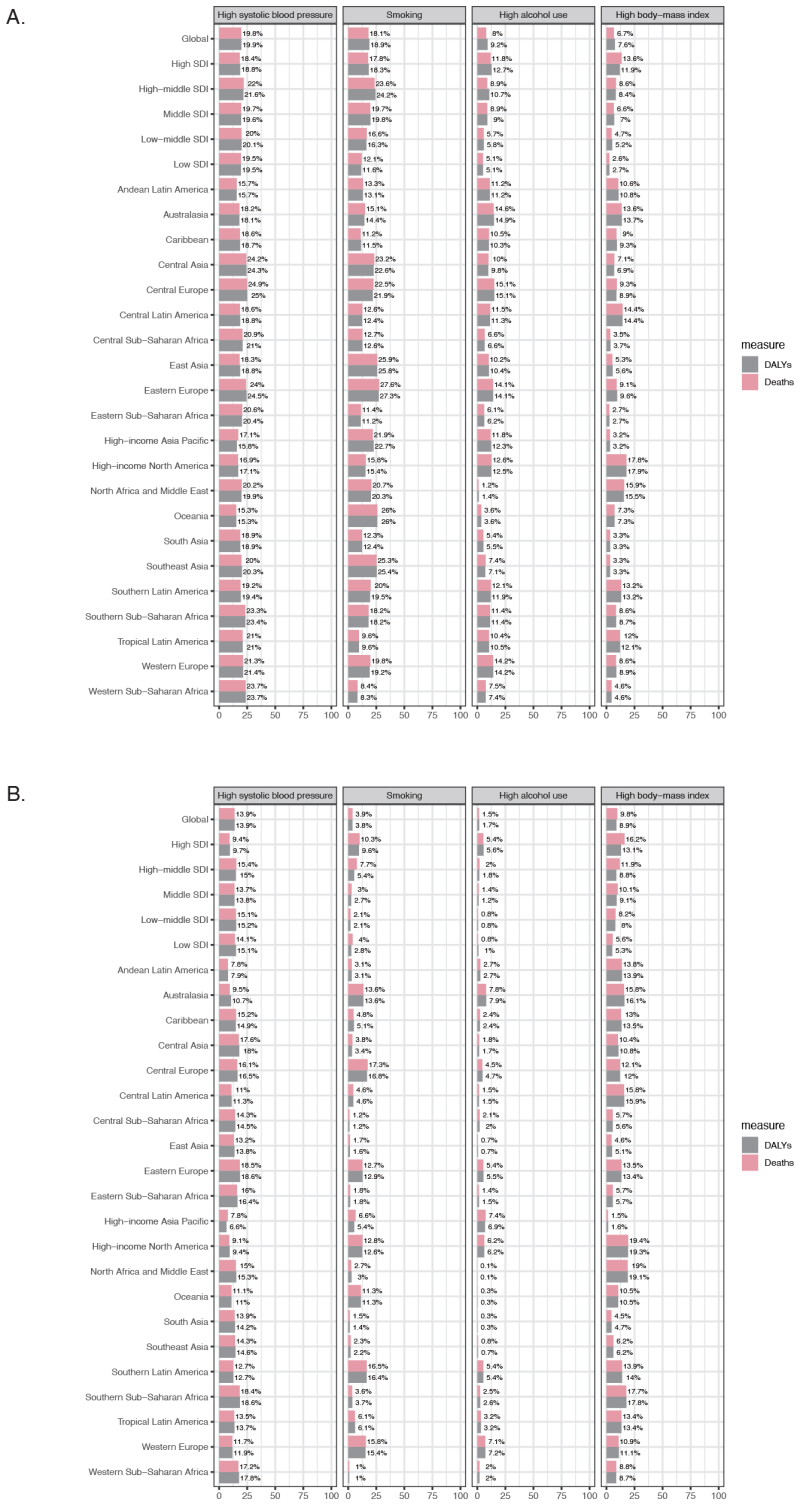
Percentage contributions of major risk factors to age-standardized death and DALYs of atrial fibrillation/atrial flutter stratified by regions and socio-demographic index, 1990–2021 in males **(A)** and females **(B)**.

We collected data on AF/AFL-related deaths and DALYs attributable to high systolic blood pressure (SBP), smoking, high alcohol use and high BMI among population aged 15–39 years, and further stratified the data by regions (**Figure 3**). SBP was the most significant contributor to deaths and DALYs due to AF/AFL in the youth in 2021, which is consistent across different SDI regions. Smoking, high alcohol use and high BMI accounted for 12.4%, 5.4% and 7.9% deaths on the global scale, respectively. Subgroup analysis in the males and females demonstrated that high SBP contributed to the highest proportions of deaths and DALYs globally in both young males and females. And the substantial risk factors were smoking in males and high BMI in females (**Figure 4**).

### High DBP is a stronger predictor of AF/AFL in the youth than high SBP

**Figure 5:**
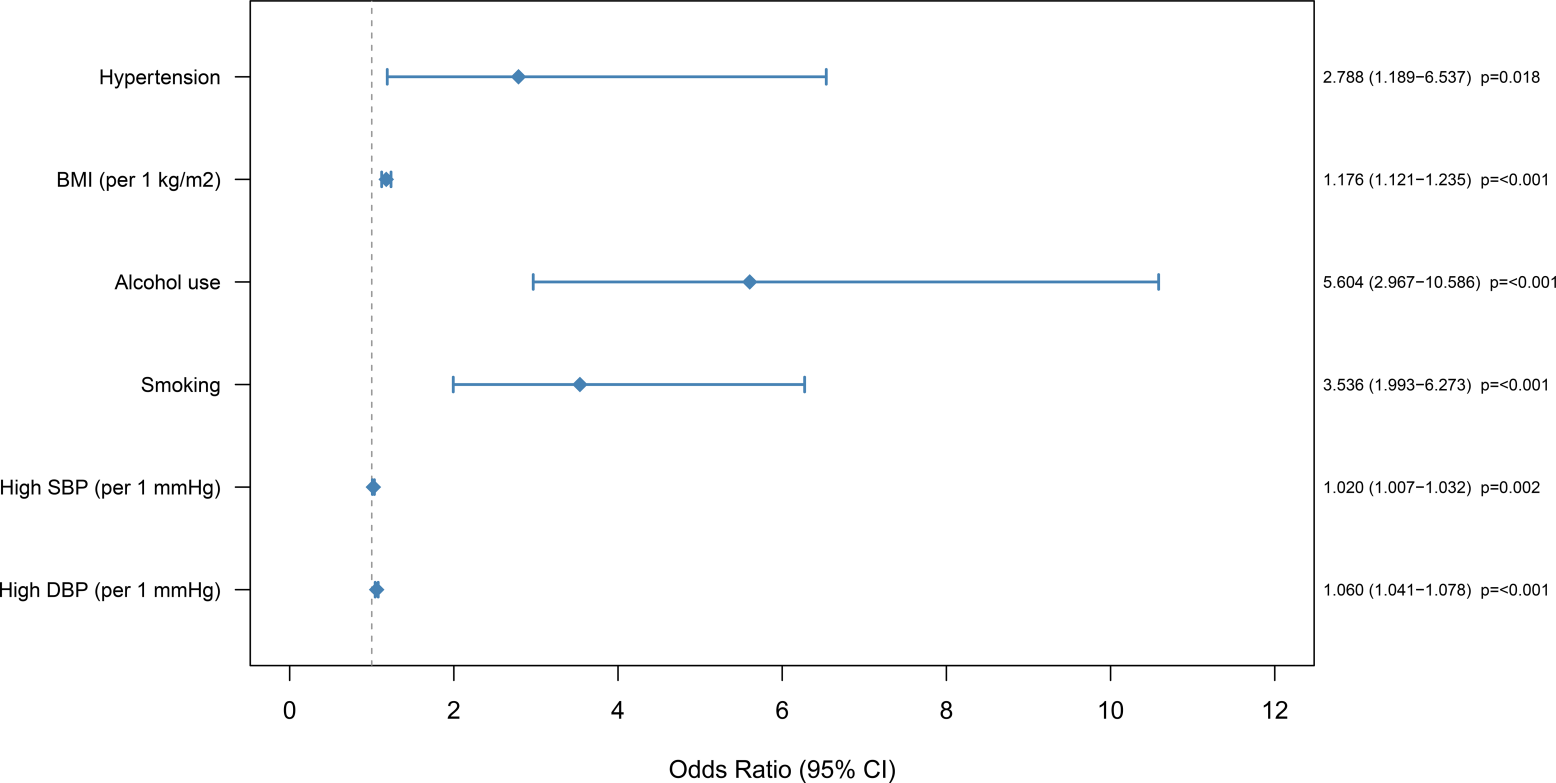
Forest plot showing the unadjusted associations between risk factors and atrial fibrillation. Odds ratios (ORs) and 95% confidence intervals (CIs) are displayed on a linear scale. The red dashed line indicates OR = 1.0 (no association). Blue symbols indicate statistically significant associations (*p* < 0.05).

We further verified the risk factors contributing to AF in a real-world cohort study. A total of 1,015 young patients were included in the analysis. The study cohort consisted of 144 patients in the AF case group (112 with PAF, 32 with CAF) and 871 patients in the PVC control group. The baseline demographic and clinical characteristics of the study population are summarized in **Table 1**. Patients in the AF group were significantly more likely to have a history of hypertension, smoking, and alcohol consumption compared to the control group. In the unadjusted logistic regression model, alcohol consumption, smoking, hypertension history, BMI, diastolic blood pressure (DBP) and SBP were all significantly associated with increased odds of AF (**Figure 5**), the adjusted model was shown in **Supplementary Figures S4**. The strongest associations were observed for alcohol consumption (OR = 5.604, 95% CI: 2.967–10.586, p < 0.001) and smoking (OR = 3.536, 95% CI: 1.993–6.273, p < 0.001). A history of hypertension was also strongly associated with AF (OR = 2.788, 95% CI: 1.189-6.537, *p* = 0.018). Furthermore, BMI (OR per 1 kg/m² = 1.176, 95% CI: 1.121-1.235, *p* < 0.001), DBP (OR per 1 mmHg = 1.060, 95% CI: 1.041–1.078, p < 0.001), and SBP (OR per 1 mmHg = 1.020, 95% CI: 1.007–1.032, p = 0.002) were all significantly associated with increased odds of AF (**Table 2**). After adjusting for age and history of heart failure, the associations for BMI, DBP, and SBP remained statistically significant, although the magnitudes of the odds ratios were slightly attenuated. However, the odds ratio for a history of hypertension, while maintaining a strong point estimate, was no longer statistically significant in the adjusted model. This is likely due to a lack of statistical power resulting from the relatively low absolute number of patients with a formal prior diagnosis of hypertension in this young cohort.

**Table 1:** Baseline characteristics of the real-world cohort stratified with atrial fibrillation status.

Characteristics	AF (N = 144)		Control group (N = 871)	*p* value
Age, year	35.0 (30.8-37.0)		30.0 (24.0-35.0)	<0.001
Female, n (%)	29/144 (20.1%)		575/871 (66.0%)	<0.001
Alcohol, n (%)	19/144 (13.2%)		23/871 (2.6%)	<0.001
Smoking, n (%)	20/144 (13.9%)		38/871 (4.4%)	<0.001
BMI, kg/m²	24.1 (21.8-26.6)		21.8 (19.7-24.0)	<0.001
Hypertension, n (%)	8/144 (5.6%)		18/871 (2.1%)	0.022
SBP, mmHg	123.0 (110.8-132.0)		118.0 (109.0-128.0)	0.0024
DBP, mmHg	80.0 (73.0-88.0)		73.0 (67.0-80.0)	<0.001
Heart failure, n (%)	7/144 (4.9%)		6/871 (0.7%)	0.001
Stroke, n (%)	1/144 (0.7%)		1/871 (0.1%)	0.2637
Coronary artery disease, n (%)	1/144 (0.7%)		0/871 (0.0%)	0.1419

AF, atrial fibrillation; BMI, body mass index; SBP, systolic blood pressure; DBP, diastolic blood pressure.

**Table 2:** Odds ratios between AF and different risk factors.

	Unadjusted		Adjusted	
	OR (95% CI)	*p* value	OR (95% CI)	*p* value
Hypertension				
Control group	Reference		Reference	
AF	2.788 (1.189-6.537)	0.018	2.273 (0.923-5.602)	0.074
BMI				
Control group	Reference		Reference	
AF	1.176 (1.121-1.235)	<0.001	1.143 (1.085-1.203)	<0.001
Alcohol use				
Control group	Reference		Reference	
AF	5.604 (2.970-10.586)	<0.001	4.430 (2.271-8.645)	<0.001
Smoking				
Control group	Reference		Reference	
AF	3.537 (1.993-6.273)	<0.001	2.848 (1.568-5.173)	<0.001
High systolic blood pressure				
Control group	Reference		Reference	
AF	1.012 (1.007-1.032)	0.002	1.019 (1.006-1.032)	0.005
High diastolic blood pressure				
Control group	Reference		Reference	
AF	1.060 (1.041-1.078)	<0.001	1.058 (1.039-1.077)	<0.001

CI, confidence interval.

**Figure 6:**
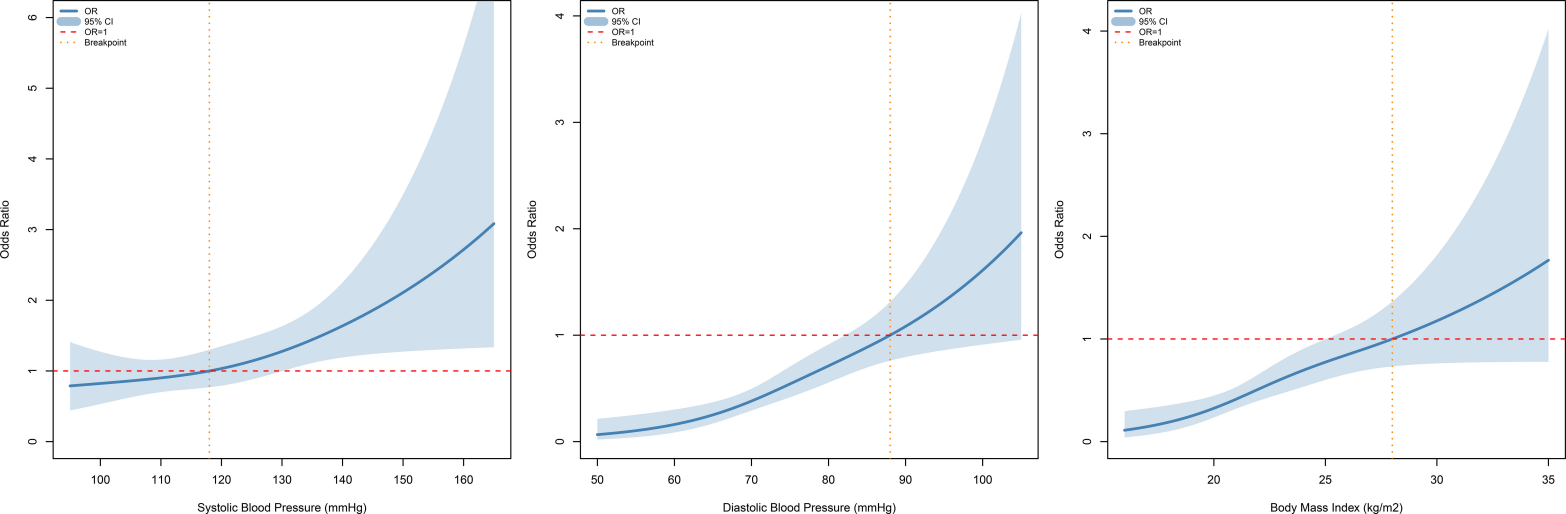
Restricted cubic spline curves showing the association between systolic blood pressure **(A)**, diastolic blood pressure **(B)**, body mass index **(C)** and the odds of atrial fibrillation. Adjusted for age and heart failure. The solid blue line represents the odds ratio, and the shaded area represents the 95% confidence interval. The red dashed line indicates OR = 1.0. The orange dotted line indicates the reference value (estimated breakpoint).

**Figure 7:**
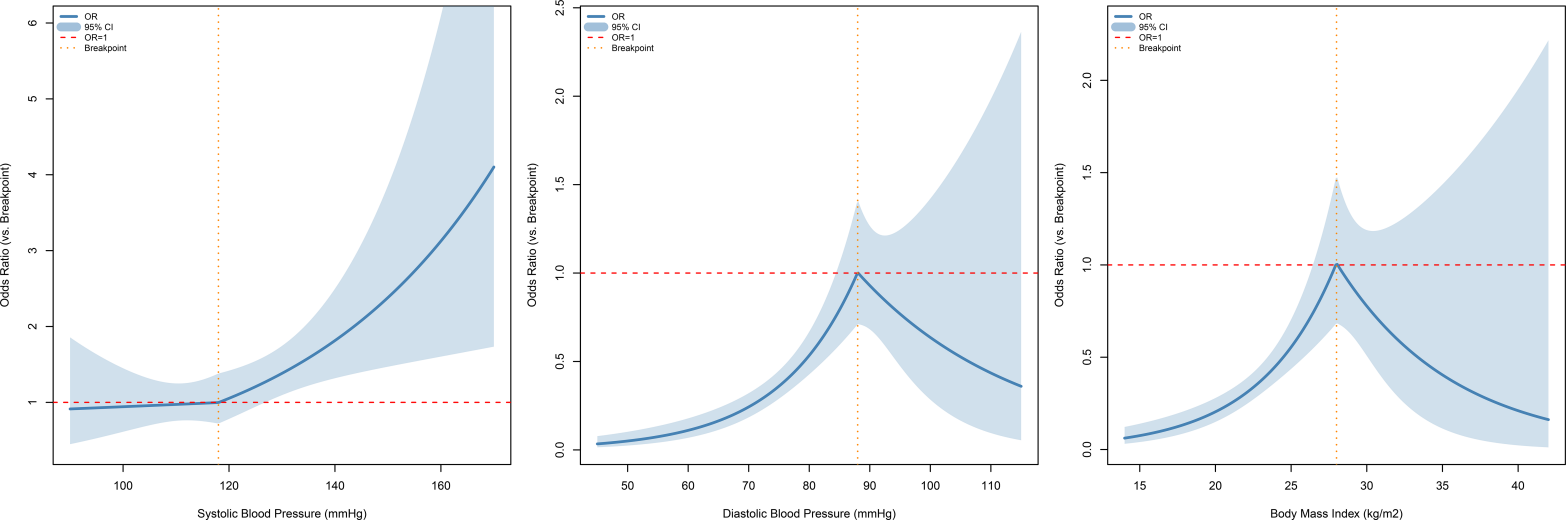
Segmented regression analysis for systolic blood pressure **(A)**, diastolic blood pressure **(B)**, body mass index **(C)**. Adjusted for age and heart failure.

To further explore the dose-response relationships, we performed restricted cubic spline (RCS) and segmented regression analyses for the continuous variables, including SBP, DBP, and BMI. In the adjusted RCS models, SBP, DBP, and BMI were significantly associated with AF overall, whereas the tests for non-linearity were not statistically significant (**Figure 6**). Segmented regression analysis was subsequently performed to identify potential breakpoints (**Figure 7**). The estimated breakpoint for SBP was 117.0 mmHg; above this value, each 1 mmHg increase in SBP was associated with a 2.7% increase in the odds of AF, although the Davies test did not support a statistically significant slope change. Significant breakpoints were identified for DBP at 88.3 mmHg (Davies test P = 0.0421) and for BMI at 28.8 kg/m^2^ (Davies test P = 0.0119). Below these breakpoints, each 1 mmHg increase in DBP and each 1 kg/m^2^ increase in BMI were associated with 8.1% and 21.4% higher odds of AF, respectively. The unadjusted models are shown in **Supplementary Figures S5** and **S6**.

### Future forecasts of global burden of AF/AFL among 15-39-year population

**Figure 8:**
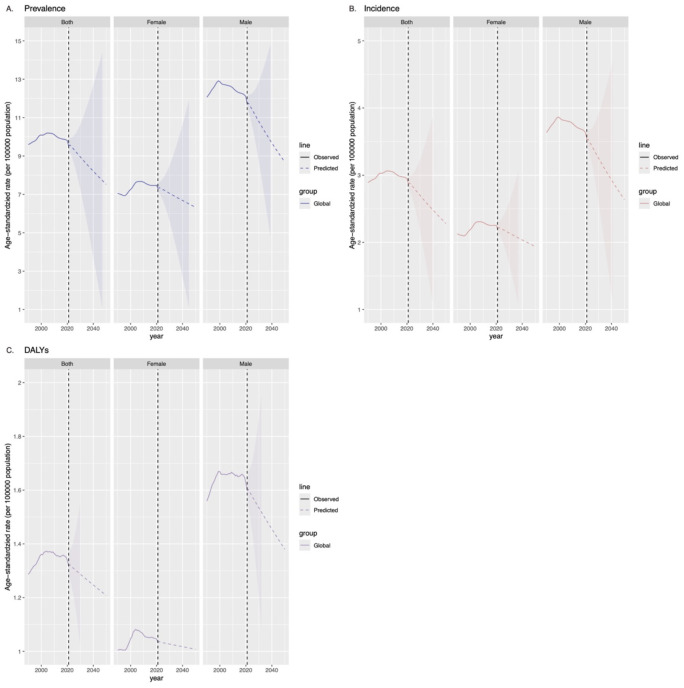
Future forecasts of global burden of atrial fibrillation/atrial flutter. **(A)** Prevalence rate. **(B)** Incidence rate. **(C)** DALYs rate.

The global burden of AF/AFL among 15-39-year population is projected to decrease from 2021 to 2050. The ASPR of AF/AFL is expected to decrease globally from approximately 9.5 per 100,000 population in 2021 to about 7.5 per 100,000 by 2050 for both sexes combined, indicating a roughly 20% decrease over 30 years (**Figure 8**). The prevalence is expected to decrease from about 11.7 per 100,000 in 2021 to 8.7 per 100,000 in 2050 in males. In contrast, the prevalence in females are relative stable, with a slight decrease from approximately 7.3 per 100,000 in 2021 to 6.3 per 100,000 in 2050. The ASIR for AF/AFL is projected to slightly decrease for both sexes combined, from about 2.9 per 100,000 population in 2021 to approximately 2.3 per 100,000 by 2050. The age-standardized DALY rate is expected to slightly decrease in males and females, with a slight decrease from 1.3 per 100,000 population in 2021 to approximately 1.2 per 100,000 by 2050. The ASPR, ASIR and age-standardized DALYs of AF/AFL are all projected to decrease in future three decades, suggesting an improvement in awareness and management of AF/AFL. Additionally, the global burden of atrial fibrillation and flutter in males is projected to be higher than that in females in our analysis, underscoring the need of sex-specific preventive strategies in the future.

## Discussion

This study uncovered an increasing global burden of atrial fibrillation and flutter (AF/AFL) in the youth from 1990 to 2021 globally. The combination of GBD study and real-world cohort study revealed that high blood pressure, smoking, drinking and high BMI contributed to increased risks of AF/AFL. The comprehensive analysis of the atrial fibrillation and flutter global burden from 1990 to 2021 in the population aged 15–39 years uncovered a landscape of the trends and disparities across regions and nations. Previous study demonstrated that age was the strongest risk factor for atrial fibrillation and flutter in comparison with hypertension, smoking and other traditional risk factors (21). Therefore, the researches about atrial fibrillation and flutter were mostly carried out in the population over 65 years old, and the current global burden of atrial fibrillation and flutter among the young population is far from clear. Additionally, atrial fibrillation and flutter was the most prevalent cardiovascular disease among the population aged 15–39 years from 1990 to 2019 (8, 22). The ASPR, ASIR, ASDR, and age-standardized DALY rate of atrial fibrillation and flutter in the youth increased from 1990 to 2021 globally. While previous analysis reported decreased ASPR and ASIR of atrial fibrillation and flutter increased from 1990 to 2019 in the total population (1, 23). The disparate trends of atrial fibrillation and flutter burden among the youth and the whole population underscore the imperative for concern to the situation of atrial fibrillation and flutter in the population aged 15 to 39 years. The global burden of atrial fibrillation and flutter among the young people exhibited socioeconomic disparities, the highest ASPR, ASIR and age-standardized DALY rates were observed in high SDI regions, while the lowest rates were found in low SDI regions. And the highest ASDR was observed in middle SDI regions, while the lowest rates were reported in high-middle SDI regions (8, 24). The higher burden in high SDI regions may result from improved disease detection rates, increased exposure to risk factors including hypertension, metabolic diseases, and inadequate secondary prevention strategies (20). There were significant decreases of ASPR, ASIR and age-standardized DALY rates in the high SDI regions from 2000 to 2012, which may benefit from improved healthcare system, more widespread use of anticoagulation for atrial fibrillation and better control of traditional risk factors. This study also found the ASPR and age-standardized DALY rates increased exponentially with increases SDI, which may be due to an improved AF/AFL awareness and brought from the well-established healthcare systems and effective public health interventions in high SDI regions.

Our results demonstrated that the ASPR, ASIR and age-standardized DALYs of atrial fibrillation and flutter in showed exponential growth at ages 30 to 39, which were consistent in the subgroup analysis stratified by sex. And the ASPR, ASIR and age-standardized DALYs of atrial fibrillation and flutter in males were higher than in females in the population aged 15–39 years in 2021. Previous GBD analysis indicated that atrial fibrillation and flutter-related incidence and prevalence were higher in males than in females, whereas the opposite results were found in atrial fibrillation and flutter-related deaths and DALYs (1, 25), which may be due to less antiarrhythmic therapy, less cardioversion and less catheter ablation in females compared to males (26, 27).

We further analyzed four strong risk factors contributing to atrial fibrillation and flutter burden, including high systolic pressure, smoking, high alcohol use and BMI (28). Our results revealed that high systolic blood pressure was the most significant contributor to deaths and DALYs due to AF/AFL in the population aged 15–39 years in 2021, which is consistent in the subgroup analysis stratified by males and females. The second most significant contributor to deaths and DALYs due to AF/AFL was smoking in young males and high BMI in young females. Hypertension and even prehypertension increases the risk of AF/AFL by inducing left atrial hypertrophy and fibrosis, thereby altering the atrial electrophysiological properties (29). Obesity promotes a pro−inflammatory state and oxidative stress, which accelerates atrial remodeling, this explains why weight loss can reverse such remodeling. Alcohol consumption reduces atrial ion channel currents, and chronic drinking leads to substrate remodeling in the left atrium, acting as the “tip of the iceberg” for alcohol-associated heart disease (30). Smoking elevates sympathetic tone, promotes inflammation, and promotes atrial fibrosis, thereby raising the risk of atrial fibrillation (31). Understanding these mechanisms aids in developing more targeted intervention strategies.

The further analysis among the real-world cohort study supported a significant and linear association between SBP and AF (OR per 1mmHg = 1.02, *p* = 0.002). While the odds ratio per unit increase appears modest, the cumulative impact of sustained elevated SBP over time is a major driver of atrial remodeling. Our findings, derived from a clinical setting, provide crucial validation that SBP is a foundational risk factor for young-onset AF, which is consistent with GBD analysis results. Below 88.3 mmHg, each 1 mmHg increase in DBP was associated with an 8.1% increase in the odds of AF. Our findings suggest that DBP exhibited a stronger association with AF than SBP. Crucially, this indicates that even DBP levels within the pre-hypertensive or high-normal range (80–89 mmHg) confer a substantial risk in young individuals, a population in which diastolic hypertension is more prevalent (32). Therefore, waiting for DBP to cross the traditional 90 mmHg threshold before intervening represents a missed opportunity for AF prevention; pre-hypertensive DBP values in young people warrant immediate clinical attention and proactive lifestyle modifications. Previous studies have demonstrated the positive relationship between SBP and AF risk. This study showed that SBP was positively associated with AF, particularly above the estimated value of 117.0 mmHg; however, the Davies test did not support a statistically significant SBP slope change, and this result should not be interpreted as a definitive clinical threshold. Further ensure the strong association between blood pressure and AF in the young population, underscoring the importance of blood pressure monitoring and individualized management according to current clinical guidelines, in the young population as a primary prevention strategy for AF.

Similarly, segmented regression identified a BMI breakpoint of 28.8 kg/m^²^. This finding is particularly salient for Asian populations, highlighting that moderate levels of adiposity, which might be considered merely overweight in Western populations, represent a significant risk for AF in this ethnic group and warrant clinical attention. The most striking finding of our study was the exceptionally strong association of lifestyle factors with AF/AFL in the young. Alcohol consumption and smoking are potent and modifiable triggers for AF/AFL. The high odds ratios in our study may reflect the relatively lower prevalence of other comorbidities in this young cohort, making the impact of these lifestyle choices more prominent. Therefore, public health initiatives and individual counseling aimed at reducing alcohol intake and promoting smoking cessation are paramount for preventing young-onset AF/AFL. These findings challenge the notion of “lone AF” in the young, suggesting that a substantial proportion of cases are associated with identifiable and modifiable risk factors including hypertension, obesity, and lifestyle choices, which calls for a paradigm shift from a primarily reactive, treatment-focused approach to a proactive, prevention-oriented strategy.

For young patients, a more proactive rhythm control strategy should be adopted to enhance quality of life and long-term prognosis. Prevention strategies for AF/AFL in the youth should adopt a multifactorial intervention approach. This study indicates that a combination of multiple healthy lifestyle factors can reduce the risk of atrial fibrillation by over 50%. Specific measures include: (1) weight management, particularly for individuals with elevated BMI; (2) intensive blood pressure control, with a paradigm shift towards earlier intervention for high-normal DBP (80–89 mmHg), aiming for targets best under 120/80 mmHg; (3) smoking cessation and limiting alcohol consumption; (4) engaging in moderate physical activity (while avoiding excessive exercise). These interventions should be adapted according to the resource availability and conditions in different regions.

In addition to traditional modifiable risk factors, emerging non-radiological indices of thoracic geometry offer promising avenues for refining risk stratification. Notably, the modified Haller index (MHI), a simple and non-invasive anthropometric parameter, has been shown to be inversely associated with asymptomatic status in AF patients (33). Integrating MHI into routine clinical assessment could complement traditional metrics like BMI and blood pressure, aiding in the more comprehensive phenotypic characterization of patients. This is particularly relevant for younger populations, where AF frequently remains underdiagnosed due to a lack of classic symptoms. Implementing MHI evaluation in routine practice is highly feasible and could enhance early detection strategies, helping to identify individuals at higher risk for asymptomatic AF and underlying structural heart disease, thereby further improving the translational impact of early intervention programs.

BAPC analysis found a slight decrease of atrial fibrillation and flutter burden in next 30 years, with ASPR decreasing globally from approximately 9.5 per 100,000 population in 2021 to about 7.5 per 100,000 by 2050, ASIR from about 2.9 per 100,000 population in 2021 to approximately 2.3 per 100,000 by 2050. The consistent results were observed in ASDR and age-standardized DALYs. These results may be attribute to increasingly improved healthcare system and popularization of disease knowledge. In addition, the decrease of atrial fibrillation and flutter in next 3 decades is relatively slight, therefore, strengthening atrial fibrillation and flutter prevention and management is still necessary in the future.

A limitation of our cohort study is the use of patients with premature ventricular contractions (PVC) as the control group. While this provided a practical comparison group from the same clinical setting, patients referred for PVC ablation may possess a distinct risk profile, such as heightened sympathetic tone or higher stress levels, that differs from a completely healthy, arrhythmia-free general population. Consequently, this could potentially influence the magnitude of the observed odds ratios, and our findings should be interpreted with this context.

In conclusion, this study revealed increasing ASPR, ASIR, ASDR, and DALY rates among individuals aged 15 to 39 years from 1990 to 2021, with marked geographic and socioeconomic disparities, underscoring the urgency for AF/AFL awareness and management strategies targeting younger populations. Both GBD data and real-world cohort study consistently identified hypertension, high BMI, smoking, and alcohol consumption as major modifiable risk factors for AF/AFL, with hypertension contributing the largest attributable risk. In the cohort study, SBP, DBP, and BMI were positively associated with AF. Segmented regression identified significant breakpoints for DBP and BMI at 88.3 mmHg and 28.8 kg/m^2^, respectively, whereas SBP showed a positive association without a statistically significant slope-change test. While BAPC analysis projected a slight reduction in AF/AFL burden, sustained efforts to improve disease awareness, strengthen health care systems, and implement early interventions remain essential. Future research should prioritize AF/AFL prevention strategies and investigate the complex interplay between genetic predisposition and lifestyle factors in disease pathogenesis.

## Data Availability

The raw data supporting the conclusions of this article will be made available by the authors, without undue reservation.
