# Supplementary material for: Assessing modifiable risk factors for atrial fibrillation/flutter in the young: a hybrid local-global study
Source: Front Endocrinol (Lausanne). 2026 May 29;17:1806013. doi: 10.3389/fendo.2026.1806013 (PMC13259807; doi:10.3389/fendo.2026.1806013)
Supplement: Supplementary file 1 [file Supplementaryfile1.docx]

**Assessing modifiable risk factors for AF/AFL in the young: a hybrid local-global study**

Ye Liu^1^, Lifeng Liu^1^, Qing Zhou^2^, Yupeng Liu ^3,4*^, Jingjing Song ^1,*^

^1^Heart Center and Beijing Key Laboratory of Hypertension，Beijing Chaoyang Hospital，Capital Medical University, Beijing, China

^2^Department of Cardiology, Beijing Hospital, National Center of Gerontology, Institute of Geriatric Medicine, Chinese Academy of Medical Sciences, No. 1, Dahua Road, Dongcheng District, Beijing 100730, China

^3^Department of Cardiology, Guangdong Provincial People's Hospital, Guangdong Academy of Medical Sciences, Southern Medical University, Guangzhou, China;

^4^Guangdong Cardiovascular Institute, Guangdong Provincial People's Hospital, Guangdong Academy of Medical Sciences, Guangzhou, China;

^*^ Corresponding author

**Address correspondence and reprint request to:**

**Jingjing Song, MD, PhD**

Heart Center and Beijing Key Laboratory of Hypertension，Beijing Chaoyang Hospital，Capital Medical University, Beijing, China

Address: No.8, Gongti South Road, Chaoyang District, Beijing, China, 100020

Email: sjjing1012@163.com

**Yupeng Liu, MD, PhD**

Department of Cardiology, Guangdong Provincial People's Hospital, Guangdong Academy of Medical Sciences, Southern Medical University, Guangzhou, China; Guangdong Cardiovascular Institute, Guangdong Provincial People's Hospital, Guangdong Academy of Medical Sciences, Guangzhou, China;

Address: No. 106, Zhongshan 2nd Road, Yuexiu District, Guangzhou City

Email: 62liuyupeng@163.com

**Figure legend**

**Figure S1.** The global disease burden of atrial fibrillation/atrial flutter for both sexes in 204 countries and territories. (A) Prevalence rate. (B) Incidence rate. (C) Death rate. (D) DALYs rate.

**Figure S2.** Trend in age-standardized prevalence(A) and DALYs rate(B) of atrial fibrillation/atrial flutter globally and for 21 GBD regions by socio-demographic index, 1990–2021.

**Figure S3.** Age-standardized prevalence rates of atrial fibrillation/atrial flutter by sex, age group, and socio-demographic index,1990-2021.

**Figure S4.** Forest plot showing the adjusted associations between risk factors and atrial fibrillation, after adjustment for age and history of heart failure. Odds ratios (ORs) and 95% confidence intervals (CIs) are displayed on a linear scale.

**Figure S5.** Restricted cubic spline curves showing the association between systolic blood pressure (A), diastolic blood pressure (B), body mass index (C) and the odds of atrial fibrillation. The solid blue line represents the odds ratio, and the shaded area represents the 95% confidence interval. The red dashed line indicates OR = 1.0. The orange dotted line indicates the reference value.

**Figure S6.** Segmented regression analysis for systolic blood pressure (A), diastolic blood pressure (B), body mass index (C).

**Figure S1**

**Figure S2**

**Figure S3**

**Figure S4**

**Figure S5**

**Figure S6**

**Table 1. Global and regional trends in atrial fibrillation/flutter: prevalence, incidence, mortality and disability-adjusted life years.**

| **Location** | **1990** | | | | | | | **2021** | | | | | | | **EAPC_95%CI** | | |  |  |
| --- | --- | --- | --- | --- | --- | --- | --- | --- | --- | --- | --- | --- | --- | --- | --- | --- | --- | --- | --- |
|  | **Number** | | | **ASR** | | | | | **Number** | | | **ASR** | | |  |  |  |  |  |
| **Prevalence** | | |  |  | | |  | | | | |  | |  | | | |  |  |
| Global | | | 187771.8 (89247.1-327175) | | | 8.6 (4.1-14.9) | | | | | 296062.9 (152846.3-499514.2) | | | | 10 (5.1-16.8) | | 0.41 (0.28 to 0.54) | | |
| High SDI | | | 56763.9 (30522.7-93629.1) | | | 16.4 (8.8-27) | | | | | 68793.7 (44593.9-98846.5) | | | | 19.5 (12.6-28) | | 0.13 (-0.15 to 0.42) | | |
| High-middle SDI | | | 43410.8 (20254-76042.8) | | | 9.6 (4.5-16.8) | | | | | 60052 (30121.3-103117.6) | | | | 13.6 (6.8-23.4) | | 0.99 (0.89 to 1.1) | | |
| Low SDI | | | 8235.3 (3523.7-15440) | | | 4.5 (1.9-8.4) | | | | | 21358.9 (9376.7-40251.5) | | | | 4.8 (2.1-9) | | 0.32 (0.27 to 0.38) | | |
| Low-middle SDI | | | 23405.3 (10157.2-43346.6) | | | 5.2 (2.2-9.6) | | | | | 49775 (21665.8-93020.5) | | | | 6.2 (2.7-11.6) | | 0.72 (0.67 to 0.77) | | |
| Middle SDI | | | 55775.2 (25998.7-100233.5) | | | 7.4 (3.5-13.3) | | | | | 95834.6 (44846.2-170707) | | | | 10.3 (4.8-18.4) | | 1.2 (1.01 to 1.4) | | |
| Andean Latin America | | | 1145.7 (523.1-2040.5) | | | 7.4 (3.4-13.2) | | | | | 2724 (1284.7-4853.9) | | | | 10.1 (4.7-17.9) | | 0.99 (0.94 to 1.05) | | |
| Australasia | | | 2172.1 (1797-2630.7) | | | 26.6 (22-32.3) | | | | | 2323.9 (1217.7-3832.7) | | | | 22.2 (11.6-36.6) | | -0.45 (-0.65 to -0.25) | | |
| Caribbean | | | 1274.1 (604.2-2255.1) | | | 8.6 (4.1-15.2) | | | | | 1967.2 (951.5-3442) | | | | 10.8 (5.2-18.9) | | 0.44 (0.22 to 0.66) | | |
| Central Asia | | | 1698.6 (781-3082.5) | | | 6 (2.7-10.8) | | | | | 3018.8 (1348-5413.3) | | | | 8.1 (3.6-14.5) | | 0.35 (0.11 to 0.59) | | |
| Central Europe | | | 4865.1 (2311.8-8461.9) | | | 10.4 (4.9-18.1) | | | | | 4824.8 (2629.6-7795.8) | | | | 13.8 (7.5-22.3) | | 0.83 (0.57 to 1.1) | | |
| Central Latin America | | | 5379.8 (2558.6-9636.6) | | | 7.9 (3.7-14.1) | | | | | 10495.8 (5037.2-18734.7) | | | | 10.4 (5-18.5) | | 0.8 (0.75 to 0.84) | | |
| Central Sub-Saharan Africa | | | 916.7 (390.9-1701.4) | | | 4.4 (1.9-8.2) | | | | | 2483.6 (1082.8-4632.8) | | | | 4.6 (2-8.6) | | 0.04 (-0.01 to 0.09) | | |
| East Asia | | | 47757.7 (22321.5-85254) | | | 8.4 (3.9-15.1) | | | | | 70495.3 (34083.2-123534.8) | | | | 14.7 (7.1-25.8) | | 1.71 (1.39 to 2.02) | | |
| Eastern Europe | | | 10497.4 (5102.8-18402.7) | | | 12.2 (5.9-21.5) | | | | | 11197.7 (5398.3-19650.1) | | | | 16.9 (8.2-29.7) | | 0.6 (0.24 to 0.96) | | |
| Eastern Sub-Saharan Africa | | | 3387.9 (1489-6326.2) | | | 4.8 (2.1-8.9) | | | | | 9643.1 (4240.6-17956.1) | | | | 5.5 (2.4-10.2) | | 0.62 (0.5 to 0.73) | | |
| High-income Asia Pacific | | | 11762.5 (6137.4-19666.5) | | | 17.4 (9.1-29.1) | | | | | 12548.5 (7914.2-18319.6) | | | | 24.8 (15.7-36.2) | | 1.9 (1.61 to 2.18) | | |
| High-income North America | | | 19698.9 (10121.4-33264.5) | | | 17.4 (8.9-29.4) | | | | | 21565.6 (16189.1-28244.6) | | | | 17.5 (13.1-22.9) | | -0.85 (-1.21 to -0.48) | | |
| North Africa and Middle East | | | 5453.5 (2330.2-10069.4) | | | 4.1 (1.7-7.5) | | | | | 16220 (7957.7-28316.9) | | | | 6.4 (3.1-11.1) | | 1.48 (1.35 to 1.61) | | |
| Oceania | | | 194 (91.1-343.6) | | | 7.3 (3.4-12.9) | | | | | 479.8 (229.5-844.5) | | | | 8.5 (4.1-15) | | 0.37 (0.3 to 0.44) | | |
| South Asia | | | 18060.9 (7389.8-35175.5) | | | 4.2 (1.7-8.1) | | | | | 38855.6 (16339-76393.3) | | | | 4.9 (2.1-9.7) | | 0.45 (0.42 to 0.48) | | |
| Southeast Asia | | | 15782.2 (7464.3-28153) | | | 8 (3.8-14.3) | | | | | 29935.1 (14208-54217.5) | | | | 10.8 (5.1-19.6) | | 0.87 (0.8 to 0.93) | | |
| Southern Latin America | | | 1455.4 (638-2573.4) | | | 7.6 (3.3-13.5) | | | | | 2426.2 (1500.9-3622.6) | | | | 9.4 (5.8-14) | | 0.65 (0.62 to 0.69) | | |
| Southern Sub-Saharan Africa | | | 1046.7 (461.8-1967.6) | | | 4.8 (2.1-9.1) | | | | | 2235.1 (979.8-4164.3) | | | | 6.6 (2.9-12.2) | | 0.72 (0.55 to 0.89) | | |
| Tropical Latin America | | | 8843.7 (4458.2-15147.9) | | | 13.8 (6.9-23.6) | | 16415.1 (8214.8-28077) | | | | | | | 18.6 (9.3-31.8) | | 2.28 (1.89 to 2.66) | | |
| Western Europe | | | 23490.1 (12656.6-38480.5) | | | 16.3 (8.8-26.7) | | | | | 27809.1 (17261.7-41221.5) | | | | 21.4 (13.3-31.8) | | 0.58 (0.16 to 1) | | |
| Western Sub-Saharan Africa | | | 2888.7 (1232-5448) | | | 4 (1.7-7.6) | | | | | 8398.7 (3622-15868.4) | | | | 4.4 (1.9-8.3) | | 0.33 (0.29 to 0.37) | | |
| **Incidence** | | |  | | |  | | | | |  | | | |  | |  | | |
| Global | | | 56754.9 (26983.3-98891.1) | | | 2.6 (1.2-4.5) | | | | | 89254.8 (46061-150643.4) | | | | 3 (1.5-5.1) | | 0.4 (0.28 to 0.52) | | |
| High SDI | | | 17115.9 (9206.6-28277.7) | | | 4.9 (2.7-8.2) | | | | | 20561.2 (13341.9-29521.8) | | | | 5.8 (3.8-8.4) | | 0.12 (-0.14 to 0.39) | | |
| High-middle SDI | | | 13083.1 (6105.7-22913.2) | | | 2.9 (1.3-5.1) | | | | | 18066.3 (9064-30973) | | | | 4.1 (2.1-7) | | 0.99 (0.9 to 1.07) | | |
| Low SDI | | | 2516.1 (1076.9-4716.5) | | | 1.4 (0.6-2.6) | | | | | 6521.8 (2863.4-12281.1) | | | | 1.5 (0.6-2.7) | | 0.32 (0.27 to 0.37) | | |
| Low-middle SDI | | | 7148.6 (3108.4-13235) | | | 1.6 (0.7-2.9) | | | | | 15067.6 (6584.7-28165.7) | | | | 1.9 (0.8-3.5) | | 0.69 (0.65 to 0.74) | | |
| Middle SDI | | | 16836.4 (7849.9-30250.4) | | | 2.2 (1-4) | | | | | 28963.3 (13557-51579) | | | | 3.1 (1.5-5.6) | | 1.16 (1 to 1.33) | | |
| Andean Latin America | | | 350.8 (160.3-624.8) | | | 2.3 (1-4) | | | | | 824.9 (389.1-1469.8) | | | | 3 (1.4-5.4) | | 0.96 (0.9 to 1.01) | | |
| Australasia | | | 654.1 (541.3-792.3) | | | 8 (6.6-9.7) | | | | | 696.8 (367-1148.7) | | | | 6.7 (3.5-11) | | -0.44 (-0.63 to -0.26) | | |
| Caribbean | | | 388.3 (184.2-687.3) | | | 2.6 (1.2-4.6) | | | | | 596.7 (288.6-1044.7) | | | | 3.3 (1.6-5.7) | | 0.4 (0.2 to 0.59) | | |
| Central Asia | | | 524.8 (240.9-952) | | | 1.8 (0.8-3.3) | | | | | 919.8 (410.8-1648.3) | | | | 2.5 (1.1-4.4) | | 0.36 (0.12 to 0.59) | | |
| Central Europe | | | 1454 (691.1-2527.5) | | | 3.1 (1.5-5.4) | | | | | 1427.6 (778.9-2305.7) | | | | 4.1 (2.2-6.6) | | 0.85 (0.62 to 1.08) | | |
| Central Latin America | | | 1649.4 (785.6-2954.5) | | | 2.4 (1.2-4.3) | | | | | 3162.9 (1518.2-5643.6) | | | | 3.1 (1.5-5.6) | | 0.75 (0.71 to 0.79) | | |
| Central Sub-Saharan Africa | | | 281.5 (120.4-522.4) | | | 1.4 (0.6-2.5) | | | | | 760.9 (332.3-1410.4) | | | | 1.4 (0.6-2.6) | | 0.03 (-0.02 to 0.08) | | |
| East Asia | | | 14237.7 (6655.4-25414.6) | | | 2.5 (1.2-4.5) | | | | | 21433.6 (10364-37571) | | | | 4.5 (2.2-7.8) | | 1.7 (1.44 to 1.96) | | |
| Eastern Europe | | | 3176 (1544-5558.6) | | | 3.7 (1.8-6.5) | | | | | 3326.9 (1605.2-5837.2) | | | | 5 (2.4-8.8) | | 0.65 (0.31 to 1) | | |
| Eastern Sub-Saharan Africa | | | 1041.6 (457.8-1944.7) | | | 1.5 (0.6-2.7) | | | | | 2951.1 (1297.8-5495) | | | | 1.7 (0.7-3.1) | | 0.6 (0.49 to 0.71) | | |
| High-income Asia Pacific | | | 3532.2 (1842.8-5898.9) | | | 5.2 (2.7-8.7) | | | | | 3707.5 (2339.1-5410.1) | | | | 7.3 (4.6-10.7) | | 1.82 (1.53 to 2.1) | | |
| High-income North America | | | 5957 (3060.9-10056.6) | | | 5.3 (2.7-8.9) | | | | | 6479.7 (4868.9-8481.1) | | | | 5.3 (4-6.9) | | -0.83 (-1.18 to -0.48) | | |
| North Africa and Middle East | | | 1671.6 (714-3085.9) | | | 1.2 (0.5-2.3) | | | | | 4852 (2380.8-8469.9) | | | | 1.9 (0.9-3.3) | | 1.43 (1.31 to 1.55) | | |
| Oceania | | | 59.6 (28-105.6) | | | 2.2 (1.1-4) | | | | | 145.9 (69.8-256.8) | | | | 2.6 (1.2-4.6) | | 0.33 (0.27 to 0.39) | | |
| South Asia | | | 5495 (2246.9-10698.3) | | | 1.3 (0.5-2.5) | | | | | 11764.3 (4947.1-23144.5) | | | | 1.5 (0.6-2.9) | | 0.43 (0.4 to 0.46) | | |
| Southeast Asia | | | 4851.1 (2294.1-8651.6) | | | 2.5 (1.2-4.4) | | | | | 9016.4 (4280.1-16323.9) | | | | 3.3 (1.5-5.9) | | 0.81 (0.76 to 0.86) | | |
| Southern Latin America | | | 441 (193.4-779.7) | | | 2.3 (1-4.1) | | | | | 732 (452.5-1093.1) | | | | 2.8 (1.8-4.2) | | 0.64 (0.61 to 0.67) | | |
| Southern Sub-Saharan Africa | | | 321.2 (141.6-603.5) | | | 1.5 (0.7-2.8) | | | | | 676.6 (296.6-1260.4) | | | | 2 (0.9-3.7) | | 0.72 (0.55 to 0.89) | | |
| Tropical Latin America | | | 2700.5 (1361.1-4627.8) | | | 4.2 (2.1-7.2) | | | | | 4896.2 (2450.6-8372.6) | | | | 5.5 (2.8-9.5) | | 2.23 (1.84 to 2.62) | | |
| Western Europe | | | 7078.7 (3815.1-11610.4) | | | 4.9 (2.6-8.1) | | | | | 8310.7 (5164.8-12309.5) | | | | 6.4 (4-9.5) | | 0.53 (0.14 to 0.93) | | |
| Western Sub-Saharan Africa | | | 888.7 (379-1676.3) | | | 1.2 (0.5-2.3) | | | | | 2572.4 (1109.2-4859.8) | | | | 1.3 (0.6-2.5) | | 0.33 (0.28 to 0.37) | | |
| **Deaths** | | |  | | |  | | | | |  | | | |  | |  | | |
| Global | | | 169 (142.4-200.4) | | | 0.00771 (0.00649-0.00914) | | | | | 289.6 (254.8-324.2) | | | | 0.00973 (0.00856-0.0109) | | 0.59 (0.48 to 0.69) | | |
| High SDI | | | 28.7 (27.2-30.4) | | | 0.00826 (0.00783-0.00877) | | | | | 32.5 (30.2-34.7) | | | | 0.00921 (0.00856-0.00982) | | 0.17 (-0.02 to 0.37) | | |
| High-middle SDI | | | 21.9 (19.5-24.6) | | | 0.00484 (0.00432-0.00544) | | | | | 24.6 (21.7-27.7) | | | | 0.00558 (0.00493-0.00628) | | 0.37 (0.3 to 0.44) | | |
| Low SDI | | | 12.2 (6.8-17.4) | | | 0.00664 (0.0037-0.00942) | | | | | 32.5 (21.5-43) | | | | 0.00723 (0.00479-0.00958) | | 0.24 (0.16 to 0.32) | | |
| Low-middle SDI | | | 35.9 (25.7-49.4) | | | 0.00793 (0.00567-0.01089) | | | | | 79.3 (66.4-92.2) | | | | 0.00988 (0.00827-0.01149) | | 0.63 (0.55 to 0.72) | | |
| Middle SDI | | | 70 (58.8-80.4) | | | 0.0093 (0.00781-0.01068) | | | | | 120.2 (103.7-135.4) | | | | 0.01296 (0.01118-0.01459) | | 0.82 (0.64 to 1) | | |
| Andean Latin America | | | 2.8 (2.2-3.5) | | | 0.01808 (0.01403-0.02235) | | | | | 4 (3.2-5) | | | | 0.01465 (0.01166-0.01858) | | -0.92 (-1.09 to -0.76) | | |
| Australasia | | | 0.4 (0.4-0.4) | | | 0.00498 (0.00449-0.00548) | | | | | 0.6 (0.5-0.6) | | | | 0.00533 (0.00466-0.00609) | | 0.03 (-0.25 to 0.31) | | |
| Caribbean | | | 2.1 (1.8-2.4) | | | 0.01391 (0.0119-0.01622) | | | | | 3 (2.4-3.7) | | | | 0.01656 (0.01341-0.0201) | | 0.42 (0.25 to 0.59) | | |
| Central Asia | | | 1.3 (1.2-1.5) | | | 0.00472 (0.0042-0.00542) | | | | | 2.1 (1.8-2.4) | | | | 0.00551 (0.00474-0.00649) | | -0.4 (-0.69 to -0.11) | | |
| Central Europe | | | 3.2 (3.1-3.4) | | | 0.00691 (0.00653-0.00736) | | | | | 1.7 (1.5-1.8) | | | | 0.00474 (0.00424-0.00526) | | -0.82 (-1.06 to -0.59) | | |
| Central Latin America | | | 7.1 (6.7-7.5) | | | 0.01039 (0.00989-0.01093) | | | | | 12.4 (11.1-13.8) | | | | 0.01224 (0.01099-0.01366) | | 0.51 (0.33 to 0.7) | | |
| Central Sub-Saharan Africa | | | 1.3 (0.7-2.1) | | | 0.00625 (0.00339-0.01001) | | | | | 3.8 (2.3-5.8) | | | | 0.00704 (0.00426-0.01077) | | 0.36 (0.19 to 0.52) | | |
| East Asia | | | 10.5 (8.2-13.2) | | | 0.00185 (0.00145-0.00233) | | | | | 9.9 (7.6-12.6) | | | | 0.00207 (0.00158-0.00263) | | -0.5 (-0.91 to -0.08) | | |
| Eastern Europe | | | 2.2 (1.7-2.8) | | | 0.00254 (0.00198-0.00321) | | | | | 2.7 (2.4-2.9) | | | | 0.00405 (0.00368-0.00443) | | 1.23 (0.92 to 1.54) | | |
| Eastern Sub-Saharan Africa | | | 5.8 (3.1-8.3) | | | 0.00812 (0.00443-0.01168) | | | | | 14.2 (8.6-21.9) | | | | 0.00812 (0.00492-0.01253) | | -0.11 (-0.3 to 0.07) | | |
| High-income Asia Pacific | | | 7.4 (6.3-8.7) | | | 0.01103 (0.0094-0.01285) | | | | | 4 (3.7-4.6) | | | | 0.00798 (0.00724-0.00903) | | -1.43 (-1.69 to -1.18) | | |
| High-income North America | | | 7.5 (7.2-7.7) | | | 0.00658 (0.00637-0.00682) | | | | | 14.6 (13.4-15.6) | | | | 0.01182 (0.0109-0.01263) | | 2.08 (1.71 to 2.45) | | |
| North Africa and Middle East | | | 10.4 (7.9-14.2) | | | 0.00779 (0.00589-0.0106) | | | | | 21.9 (18.4-27.2) | | | | 0.00863 (0.00722-0.0107) | | 0.45 (0.27 to 0.63) | | |
| Oceania | | | 2.1 (1.2-3.1) | | | 0.07775 (0.04475-0.11781) | | | | | 5.3 (3.3-7.8) | | | | 0.09396 (0.05928-0.1377) | | 0.44 (0.33 to 0.54) | | |
| South Asia | | | 22.6 (13.3-33.6) | | | 0.00523 (0.00307-0.0078) | | | | | 54.8 (42.7-66.9) | | | | 0.00692 (0.0054-0.00846) | | 0.91 (0.79 to 1.03) | | |
| Southeast Asia | | | 47.8 (35.9-58.3) | | | 0.02427 (0.01825-0.02959) | | | | | 86.4 (69.7-104.2) | | | | 0.03117 (0.02514-0.03759) | | 0.57 (0.37 to 0.78) | | |
| Southern Latin America | | | 1.9 (1.7-2.1) | | | 0.01002 (0.00913-0.01116) | | | | | 2.4 (2.2-2.7) | | | | 0.00935 (0.00846-0.01031) | | 0.43 (0.14 to 0.72) | | |
| Southern Sub-Saharan Africa | | | 6.6 (5.5-7.7) | | | 0.03054 (0.02541-0.03558) | | | | | 10.7 (8.7-13.3) | | | | 0.03137 (0.02554-0.03901) | | -0.05 (-0.92 to 0.82) | | |
| Tropical Latin America | | | 8.3 (7.9-8.8) | | | 0.01295 (0.01228-0.01367) | | | | | 15.8 (15.1-16.7) | | | | 0.0179 (0.01708-0.01889) | | 0.92 (0.79 to 1.04) | | |
| Western Europe | | | 14.5 (13.7-15.3) | | | 0.01004 (0.00948-0.01063) | | | | | 10.5 (10-11.2) | | | | 0.0081 (0.00768-0.00863) | | -0.8 (-1.03 to -0.56) | | |
| Western Sub-Saharan Africa | | | 3.3 (2.4-4.3) | | | 0.00459 (0.0034-0.00597) | | | | | 8.9 (5.9-11.5) | | | | 0.00464 (0.00309-0.00603) | | -0.04 (-0.16 to 0.09) | | |
| **Disability-adjusted life years** | | | |  | |  | | | | |  | | | |  | |  | | |
| Global | | | 25233.5 (16478.8-39860.8) | | | 1.2 (0.8-1.8) | | | | | 40963.1 (27555.3-62914.7) | | | | 1.4 (0.9-2.1) | | 0.48 (0.36 to 0.59) | | |
| High SDI | | | 6366.4 (3970.6-10256.7) | | | 1.8 (1.1-3) | | | | | 7573 (5134.5-11163.4) | | | | 2.1 (1.5-3.2) | | 0.13 (-0.11 to 0.37) | | |
| High-middle SDI | | | 4905.1 (2728.9-8249.5) | | | 1.1 (0.6-1.8) | | | | | 6462.2 (3749.6-10964.3) | | | | 1.5 (0.9-2.5) | | 0.85 (0.78 to 0.93) | | |
| Low SDI | | | 1368.2 (808.8-2128) | | | 0.7 (0.4-1.2) | | | | | 3590 (2191.3-5544) | | | | 0.8 (0.5-1.2) | | 0.29 (0.23 to 0.34) | | |
| Low-middle SDI | | | 3987.4 (2551.9-6026.1) | | | 0.9 (0.6-1.3) | | | | | 8588.5 (5926.3-12922.5) | | | | 1.1 (0.7-1.6) | | 0.67 (0.61 to 0.72) | | |
| Middle SDI | | | 8572.2 (5799.2-12966.4) | | | 1.1 (0.8-1.7) | | | | | 14699.4 (10077-22439.8) | | | | 1.6 (1.1-2.4) | | 1.02 (0.86 to 1.19) | | |
| Andean Latin America | | | 249 (177.6-340.8) | | | 1.6 (1.1-2.2) | | | | | 441.1 (288-662.2) | | | | 1.6 (1.1-2.4) | | -0.09 (-0.19 to 0.02) | | |
| Australasia | | | 203 (127.6-303) | | | 2.5 (1.6-3.7) | | | | | 227.1 (109.1-432.2) | | | | 2.2 (1-4.1) | | -0.36 (-0.57 to -0.15) | | |
| Caribbean | | | 221.2 (156.8-328.5) | | | 1.5 (1.1-2.2) | | | | | 332 (226.9-489.6) | | | | 1.8 (1.2-2.7) | | 0.41 (0.26 to 0.56) | | |
| Central Asia | | | 219.5 (127.1-384.2) | | | 0.8 (0.4-1.4) | | | | | 371.3 (210.3-637.2) | | | | 1 (0.6-1.7) | | 0.1 (-0.13 to 0.34) | | |
| Central Europe | | | 590.4 (369.8-968.8) | | | 1.3 (0.8-2.1) | | | | | 505.1 (289.7-838.8) | | | | 1.4 (0.8-2.4) | | 0.46 (0.23 to 0.69) | | |
| Central Latin America | | | 842.7 (572.3-1250) | | | 1.2 (0.8-1.8) | | | | | 1546 (1013.7-2381.3) | | | | 1.5 (1-2.4) | | 0.65 (0.59 to 0.71) | | |
| Central Sub-Saharan Africa | | | 147.2 (75.1-245.1) | | | 0.7 (0.4-1.2) | | | | | 412.8 (228.5-690.4) | | | | 0.8 (0.4-1.3) | | 0.22 (0.14 to 0.3) | | |
| East Asia | | | 4641.7 (2225-8334.4) | | | 0.8 (0.4-1.5) | | | | | 6586.3 (3248.4-11954.7) | | | | 1.4 (0.7-2.5) | | 1.49 (1.2 to 1.79) | | |
| Eastern Europe | | | 1018.8 (515.6-1789.1) | | | 1.2 (0.6-2.1) | | | | | 1102.5 (593.6-2009.8) | | | | 1.7 (0.9-3) | | 0.71 (0.41 to 1.01) | | |
| Eastern Sub-Saharan Africa | | | 597.8 (348.1-896.1) | | | 0.8 (0.5-1.3) | | | | | 1584.8 (928.2-2440.7) | | | | 0.9 (0.5-1.4) | | 0.26 (0.12 to 0.4) | | |
| High-income Asia Pacific | | | 1398.8 (852.9-2220) | | | 2.1 (1.3-3.3) | | | | | 1283.5 (800.7-1917.5) | | | | 2.5 (1.6-3.8) | | 1.1 (0.9 to 1.3) | | |
| High-income North America | | | 2066.1 (1200.5-3499.8) | | | 1.8 (1.1-3.1) | | | | | 2587.5 (1902.2-3526.4) | | | | 2.1 (1.5-2.9) | | -0.18 (-0.51 to 0.14) | | |
| North Africa and Middle East | | | 1043.6 (679.2-1563.4) | | | 0.8 (0.5-1.2) | | | | | 2578.3 (1759.8-3886.6) | | | | 1 (0.7-1.5) | | 0.93 (0.78 to 1.09) | | |
| Oceania | | | 128.8 (78-187.2) | | | 4.8 (2.9-7) | | | | | 328.7 (222-465.3) | | | | 5.8 (3.9-8.3) | | 0.43 (0.33 to 0.52) | | |
| South Asia | | | 2815.7 (1641.3-4471.7) | | | 0.7 (0.4-1) | | | | | 6361.3 (4081.6-10126.9) | | | | 0.8 (0.5-1.3) | | 0.65 (0.6 to 0.71) | | |
| Southeast Asia | | | 3953.1 (2830-5335.9) | | | 2 (1.4-2.7) | | | | | 7260.8 (5427.4-9658.4) | | | | 2.6 (2-3.5) | | 0.67 (0.51 to 0.83) | | |
| Southern Latin America | | | 227.6 (148.4-365.4) | | | 1.2 (0.8-1.9) | | | | | 341 (218.4-508.1) | | | | 1.3 (0.8-2) | | 0.55 (0.42 to 0.68) | | |
| Southern Sub-Saharan Africa | | | 450.8 (359.6-558) | | | 2.1 (1.7-2.6) | | | | | 766.6 (605-1000.5) | | | | 2.3 (1.8-2.9) | | 0.09 (-0.59 to 0.78) | | |
| Tropical Latin America | | | 1197.2 (795.3-1887.7) | | | 1.9 (1.2-2.9) | | | | | 2233.1 (1484.6-3507.5) | | | | 2.5 (1.7-4) | | 1.83 (1.59 to 2.06) | | |
| Western Europe | | | 2791.3 (1746.3-4487.2) | | | 1.9 (1.2-3.1) | | | | | 2914.7 (1829.7-4516.2) | | | | 2.2 (1.4-3.5) | | 0.23 (-0.13 to 0.59) | | |
| Western Sub-Saharan Africa | | | 429.3 (258-684.4) | | | 0.6 (0.4-1) | | | | | 1198.7 (733.6-1995.2) | | | | 0.6 (0.4-1) | | 0.14 (0.1 to 0.18) | | |

**ASR, age-standardized rates; EAPC, estimated annual percentage changes.**
